# Serum vitamin C levels and risk of non-alcoholic fatty liver disease: results from a cross-sectional study and Mendelian randomization analysis

**DOI:** 10.3389/fnut.2023.1162031

**Published:** 2023-05-12

**Authors:** Hui Wu, Jiang-Long Guo, Jing-Jiong Yao, Jia-Jun Yu, Run-Yu Xia, Wei-Qing Huang, Xuan Tang, Guang-Ming He

**Affiliations:** ^1^Department of Radiology, The Second Affiliated Hospital of Guangzhou Medical University, Guangzhou, China; ^2^Department of Medical Imaging, The Second Clinical School of Guangzhou Medical University, Guangzhou, China; ^3^Department of Clinical Medicine, The Second Clinical School of Guangzhou Medical University, Guangzhou, China; ^4^Department of Anesthesiology, The Second Clinical School of Guangzhou Medical University, Guangzhou, China; ^5^Guangzhou Medical University, Guangzhou, China

**Keywords:** vitamin C, Mendelian randomization, NHANES, NAFLD, cross-sectional study

## Abstract

**Background and aims:**

Vitamin C, as an antioxidant, may play a role in the treatment of NAFLD. This research aimed to investigate the association of serum vitamin C levels with the risk of NAFLD and to further examine the causal relationship by Mendelian randomization (MR) method.

**Methods:**

The cross-sectional study selected 5,578 participants of the National Health and Nutrition Examination Survey (NHANES), 2005–2006 and 2017–2018. The association of serum vitamin C levels with NAFLD risk was evaluated under a multivariable logistic regression model. A two-sample MR study, using genetic data from large-scale genome-wide association studies (GWAS) of serum vitamin C levels (52,014 individuals) and NAFLD (primary analysis: 1,483 cases /17,781 controls; secondary analysis: 1,908 cases/340,591 controls), was conducted to infer causality between them. The inverse-variance-weighted (IVW) was applied as the main method of MR analysis. A series of sensitivity analyzes were used to evaluate the pleiotropy.

**Results:**

In the cross-sectional study, results showed that Tertile 3 group (Tertile 3: ≥1.06 mg/dl) had a significantly lower risk (OR = 0.59, 95% CI: 0.48 ~ 0.74, *p* < 0.001) of NAFLD than Tertile 1 group (Tertile 1: ≤0.69 mg/dl) after full adjustments. In regard to gender, serum vitamin C was protective against NAFLD in both women (OR = 0.63, 95% CI: 0.49 ~ 0.80, *p* < 0.001) and men (OR = 0.73, 95% CI: 0.55 ~ 0.97, *p* = 0.029) but was stronger among women. However, in the IVW of MR analyzes, no causal relationship between serum vitamin C levels and NAFLD risk was observed in the primary analysis (OR = 0.82, 95% CI: 0.47 ~ 1.45, *p* = 0.502) and secondary analysis (OR = 0.80, 95% CI: 0.53 ~ 1.22, *p* = 0.308). MR sensitivity analyzes yielded consistent results.

**Conclusion:**

Our MR study did not support a causal association between serum vitamin C levels and NAFLD risk. Further studies with greater cases are warranted to confirm our findings.

## 1. Introduction

Non-alcoholic fatty liver disease (NAFLD) is the most common chronic liver disorder today with a worldwide incidence of approximately 25% (1) and is one of the main reasons for liver cirrhosis in adults in the United States (2). NAFLD refers to steatosis in more than 5% of hepatocytes that is related with metabolic risk factors (particularly abnormal obesity and type II diabetes) and without excessive alcoholic beverage use (30 g/d in men, 20 g/d in women) and other chronic liver diseases (3). The pathogenesis of NAFLD is poorly comprehended. The “multiple hits” theory proposes that various factors, including insulin resistance, gut microbes, genetic susceptibility, and epigenetic factors, can jointly drive the development of NAFLD (4). Among them, oxidative stress is considered as an important cause of inducing “multiple hits” (5). There are no effective drugs for the cure of NAFLD passed by Food and Drug Administration (FDA). Vitamin C, as a commonly used antioxidant, has aroused researchers’ interest.

Vitamin C can react with free radicals and oxides form less reactive products, to protect cells from the damage of these substances in normal physiological reaction or disease so that it is often considered the preferred antioxidant (6). In genetics, vitamin C has a certain degree of epigenetic regulation function. Related studies have found that when humans lack vitamin C, it may disrupt the methylation-demethylation response of DNA and histones, resulting in phenotype changing (7). Vitamin C as an antioxidant, it is widely used in the cure and prevention of diverse diseases, such as the treatment of COVID-19 (8), prevention of skin aging (9), prevention of macular degeneration (10), adjuvant treatment of Periodontitis (11), etc. Xie et al. found a negative association between serum vitamin C levels and NAFLD (12). However, their study only selected the data from the National Health and Nutrition Examination Survey (NHANES) database from 2017 to 2018, and their study was limited only to cross-sectional studies and did not deeply explore them genetically. At present, there are few clinical trials on the effect of vitamin C on NAFLD, through searching *Home–ClinicalTrials.gov* , only Professor Georgios Papatheodoridis from the University of Athens is conducting the study, and the clinical trial results have not been released.

Therefore, to assess the effects of plasma vitamin C concentration on NAFLD risk, we first conducted an observational study with data based the US population from NHANES database (13). Furthermore, we applied Mendelian randomization (MR) approach to assess the causal correlation of circulating vitamin C levels with the risk of NAFLD. This approach could infer causality with genetically instrumental variables (IVs). Since the genetic variants are assigned arbitrarily during gamete formation under no effect from environmental and lifestyle factors, MR analysis is less susceptible to the biases from reverse causality and uncontrolled confounders, which instead were commonly found in conventional observational studies (14, 15).

## 2. Material and methods

### 2.1. Cross-sectional study

#### 2.1.1. Study design and participants

We analyzed the data from the NHANES 2005–2006 and 2017–2018. A cross-sectional study was designed to assess health and nutrition status of Americans’ adults and children with the nationally representative study. Participants in this study were interviewed about demographics, socioeconomic, dietary habits, and health. The NHANES is a major program of the National Center for Health Statistics (NCHS) and it was approved by the Research Ethics Review Board at the NCHS. A total of 19,602 participants participated the survey during 2005–2006 and 2017–2018. However, we excluded 5,560 samples with unknown serum vitamin C levels and 8,464 samples with unknown NAFLD results. Therefore, the final analytic sample included 5,578 participants with complete serum vitamin C levels and NAFLD results.

#### 2.1.2. The vitamin C levels in serum

To analyze serum specimens, the blood samples were collected, processed, stored, and delivered to Atlanta, GA’s Division of Laboratory Sciences, National Center for Environmental Health, Centers for Disease Control and Prevention. NHANES quality assurance and quality control processes (QA/QC) meet Clinical Laboratory Improvement Act (CLIA) criteria. Isocratic ultra-high performance liquid chromatography (UPLC) with electrochemical detection at 450 mV was used to quantify vitamin C in serum (range 200 nA). The peak area of vitamin C in the unknown is compared to the peak area of a known amount in a calibrator solution for quantification. The calibration solution is corrected by comparing the peak area of the internal standard with that of the unknown.

#### 2.1.3. Non-alcoholic fatty liver disease

Liver steatosis and fibrosis were determined by using the controlled attenuation parameter (CAP) from the vibration-controlled transient elastography (VCTE). NAFLD was defined by controlled attenuation parameter (CAP) scores of 248 dB/m without excessive alcohol consumption and viral hepatitis.

#### 2.1.4. Statistical analysis

R version 4.2.2 was used for all analyzes. For categorical variables, counts and proportions were employed, while means and standard deviations were used for continuous data. And then, the two-sided *p* < 0.05 was regarded as statistical significance. Serum vitamin C was divided into tertiles, and the tertile 1 (T1) was used as the reference. This research constructed logistic regression models to evaluate the associations of serum vitamin C concentrations with NAFLD risk.

Four Logistic regression models were constructed to assess the relationship. The first model did not adjust for its associated covariates, roughly estimating the association. The second model was further adjusted for age, gender, and race. The third model controlled for marital status, BMI, PIR, education level, smoking status, CVD, PA total time, and PA total MET. The fourth model was adjusted for DM and hypertension. The detailed descriptions of the covariates adjusted for are shown in Supplementary text. We also examined whether the association differed between male and female. These models were specified as follows:

*Crude model: not adjusted.*

*Model 1: adjusted for age, gender and race.*

*Model 2: Model 1 + Marital status, BMI, PIR, educational level, smoking status, CVD, PA total time and PA total MET.*

*Model 3: Model 2 + DM and Hypertension.*

### 2.2. Mendelian randomization study

#### 2.2.1. Study design

**Figure 1:**
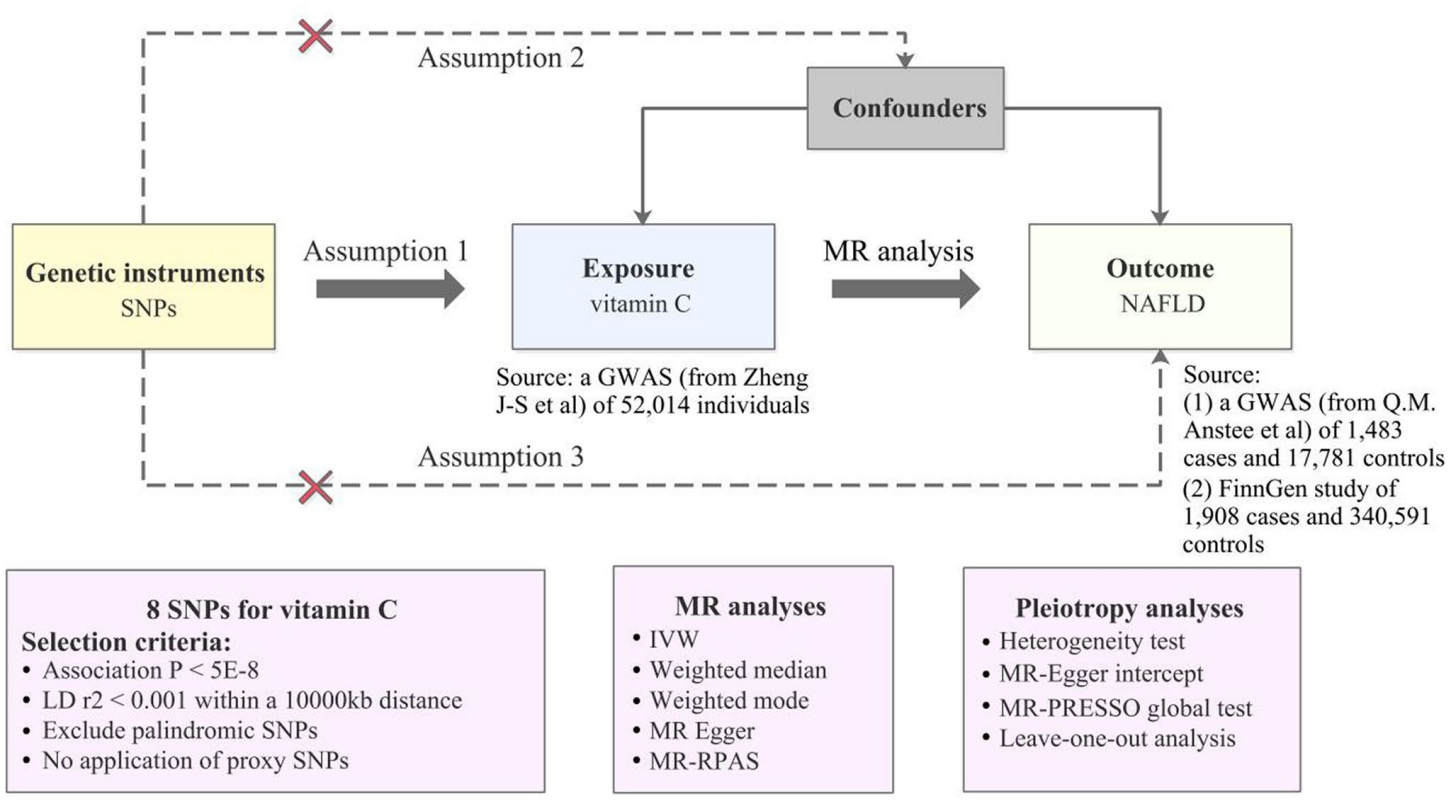
An overview of MR analysis. SNPs, single-nucleotide polymorphisms; GWAS, genome-wide association studies; MR, Mendelian randomization; LD, linkage disequilibrium; IVW, inverse variance weighted; MR-RAPS, MR-Robust Adjusted Profile Score; NAFLD, non-alcoholic fatty liver disease; MR-PRESSO, MR-Pleiotropy RESidual Sum and Outlier.

Figure 1 shows an overview of the present MR study. To obtain valid causal estimates, IVs in the MR model need to satisfy the three assumptions (Figure 1): 1 genetic variants are significantly associated with the serum vitamin C levels; (2) they are not influenced by potential confounders; and (3) they influence NAFLD only *via* serum vitamin C levels.

#### 2.2.2. Outcome data sources

Summary-level GWAS statistics of NAFLD were obtained from a largely European population-based meta-analysis by Anstee et al., with 1,483 cases and 17,781 controls, as a primary analysis (16) and the FinnGen study with 1908 cases and 340,591 controls of European descent, as a secondary analysis (17, 18). In the Anstee et al. GWAS, patients with NAFLD were enlisted from clinics at some top European tertiary liver centers, and after undergoing a liver biopsy, biopsy tissues were evaluated by liver pathologists in accordance with established standards (16). NAFLD Cases in FinnGen consortium were diagnosed under the standard of International Classification of Disease code K76.0. Detailed information on data sources is demonstrated in Supplementary Table S1.

#### 2.2.3. Genetic instrument selection

Genetic instruments (SNPs) of plasma vitamin C levels were obtained from the most recent meta-analysis of genome-wide association study (GWAS) with up to 52,018 European participants included, which identified 11 lead SNPs explaining approximately 1.87% of the variance of circulating vitamin C (19). There were four studies involved in this large-scale GWAS: the Fenland study, European Prospective Investigation into Cancer and Nutrition (EPIC)-InterAct study, EPIC-Norfolk study, and EPIC-CVD study (19). Rs174547 located in the FADS1 gene was directly removed in the analysis for encoding plasma phospholipid fatty acids synthesis-associated enzyme (19), avoiding related pleiotropic effects on our results. Additionally, we also exclude rs12524110 identified as a palindromic SNP in the harmonization process. The remaining 9 SNPs were selected as IVs in this MR analysis (Supplementary Table S2). The specific selection criteria are illustrated in Figure 1 and Supplementary Table S2.

#### 2.2.4. Statistical analysis

The main MR analysis was carried out by employing inverse-variance weighted (IVW) method under a random-effects model, which combined the Wald ratios by dividing the SNP–outcome effect by the SNP–exposure effect (20). The results were presented as odds ratios (ORs) for the risk of NAFLD per one standard deviation rise in the serum vitamin C levels. We also used MR-Egger (21), MR-RAPS (22), Weighted mode (23) and Weighted-median (24) as sensitivity analyzes to validate the results from IVW. To reduce possible pleiotropic biases, we firstly searched for and remove a confounding SNP (rs174547) using the PhenoScanner V2 database^1^ (25). The potential horizontal pleiotropy was evaluated using MR-Egger regression based on its intercept term, of which the value close to zero (*p* > 0.05) indicates no evidence for the existence of directional pleiotropy (21). Moreover, MR Pleiotropy RESidual Sum and Outlier (PRESSO) global test was utilized to remove any outliers leading to horizontal pleiotropy (26). The Cochran Q test for the IVW model was used to detect heterogeneity (27). Specifically, no heterogeneity was suggested when the Cochran Q test value of p was >0.05 and I^2^ was <25%. Besides, the Rucker Q’ test for the MR-Egger model was applied, with the difference value of Q - Q’ calculated. In detail, the value of *p* >0.05 in Q–Q’ shows that no potential pleiotropy was examined, and provides strong support that IVW model could better fit in the MR analyzes (27). In addition, the leave-one-out analysis was applied to examine whether any single SNP significantly drove the IVW estimates. Finally, we performed MR Steiger filtering test to exclude “FALSE” direction in case of possible reverse causation of plasma vitamin C on NAFLD (28). All MR analyzes were performed from the ‘TwoSampleMR’ (version 0.5.6) and ‘MR-PRESSO’ package (26) in R software 4.2.2. *p*-value <0.05 was considered as statistical significance.

We calculated F-statistic, an indicator of instrument strength measure, and the F-statistic above 10 reveals a low chance of weak instrument bias in our MR analysis (29).

## 3. Results

### 3.1. Cross-sectional study

#### 3.1.1. Basic characteristic of study participants

Overall characteristics of the study subjects by gender are summarized in Table 1. There are 2,819 women (50.54%) and 2,759 men (49.46%) in the final analytic sample. There were 35.2% NAFLD and 64.8% not NAFLD among all samples. Except for age, race, marital status, PIR, and hypertension, there were statistically significant differences between males and females in the remaining results (*p* < 0.05). Table 1 shows that serum vitamin C levels are higher in women than in men, but the prevalence of NAFLD is higher than in men.

**Table 1:** Basic characteristic of study participants.

Variables	Total (*n* = 5,578)	Female (*n* = 2,819)	Male (*n* = 2,759)	*p*-value
Age (year), Median(IQR)	46.0 (26.0, 62.0)	46.0 (27.0, 62.0)	46.0 (25.0, 63.0)	0.837
Race, *n* (%)				0.22
Mexican American	822 (14.7)	421 (14.9)	401 (14.5)	
Non-Hispanic black	1,242 (22.3)	645 (22.9)	597 (21.6)	
Non-Hispanic white	1899 (34.0)	940 (33.3)	959 (34.8)	
Other Hispanic	520 (9.3)	280 (9.9)	240 (8.7)	
Other race-including multiracial	1,095 (19.6)	533 (18.9)	562 (20.4)	
Marry, *n* (%)				0.074
No/Unknown	845 (18.3)	407 (17.3)	438 (19.4)	
Yes	3,766 (81.7)	1942 (82.7)	1824 (80.6)	
BMI(kg/m^2^), *n* (%)				<0.001
<25	1786 (32.3)	925 (33)	861 (31.5)	
25–29.9	1,692 (30.6)	769 (27.5)	923 (33.8)	
>30	2053 (37.1)	1,105 (39.5)	948 (34.7)	
PIR, Median(IQR)	2.1 (1.1, 4.0)	2.0 (1.1, 4.0)	2.1 (1.2, 4.0)	0.14
Education Level, *n* (%)				<0.001
Less than high school	1,695 (30.4)	803 (28.5)	892 (32.4)	
High school graduation	1,214 (21.8)	596 (21.2)	618 (22.4)	
College or above	2,661 (47.8)	1,416 (50.3)	1,245 (45.2)	
Smoke, *n* (%)				<0.001
Never	2,871 (59.3)	1700 (69)	1,171 (49.3)	
Former	1,124 (23.2)	405 (16.5)	719 (30.2)	
Now	844 (17.4)	357 (14.5)	487 (20.5)	
CVD, *n* (%)				<0.001
No	4,075 (88.9)	2,132 (91)	1943 (86.6)	
Yes	511 (11.1)	210 (9)	301 (13.4)	
PA total time (min/week), Median (IQR)	660.0 (240.0, 1915.0)	480.0 (180.0, 1305.0)	855.0 (300.0, 2520.0)	<0.001
PA total MET, Median (IQR)	2640.0 (960.0, 7660.0)	1920.0 (720.0, 5220.0)	3420.0 (1200.0, 10080.0)	<0.001
DM, *n* (%)				<0.001
No	4,208 (75.4)	2,180 (77.3)	2028 (73.5)	
Yes	1,370 (24.6)	639 (22.7)	731 (26.5)	
Hypertension, *n* (%)				0.082
No	3,442 (62.0)	1772 (63.2)	1,670 (60.9)	
Yes	2,107 (38.0)	1,034 (36.8)	1,073 (39.1)	
Vitamin C, mg/dl	0.9 ± 0.5	1.0 ± 0.6	0.8 ± 0.5	<0.001
Non-alcoholic fatty liver disease, *n* (%)				<0.001
No	3,612 (64.8)	1731 (61.4)	1881 (68.2)	
Yes	1966 (35.2)	1,088 (38.6)	878 (31.8)	

Number and proportion were presented for categorical variables, mean and standard deviation were presented for continuous variables.

IQR, interquartile range; BMI, body mass index; PIR, poverty income ratio; CVD, cardiovascular disease; PA, physical activity; MET, metabolic equivalent of task; DM, diabetes mellitus.

#### 3.1.2. Associations between NAFLD and serum vitamin C (all participants)

Table 2 illustrates the association between the serum level of vitamin C and the risk of NAFLD. The results indicated that the serum vitamin C levels had the weakest protective correlation for the risk of NAFLD in the crude model (OR: 0.71, 95% CI: 0.64 ~ 0.80, *p* < 0.001). After adjusting for age, gender, and race in the model 1, the association magnitude increased slightly (OR: 0.63, 95% CI: 0.55 ~ 0.71, *p* < 0.001). The model 2 on the basis of the model 1, which controlled marital status, BMI, PIR, education level, smoking status, CVD, PA total time, and PA total MET (OR: 0.66, 95% CI: 0.55 ~ 0.79, *p* < 0.001). The model 3 adjusted for diabetes and hypertension, the association was stable (OR = 0.66, 95% CI: 0.55 ~ 0.80, *p* < 0.001).

**Table 2:** Association between serum vitamin C level and NAFLD (All Participants), NHANES 2005–2006, 2017–2018.

Models	*n*	Nonalcoholic fatty liver	
		OR(95%CI)	*P*-value
Crude model	5,578	0.71 (0.64 ~ 0.80)	<0.001
Model 1	5,578	0.63 (0.55 ~ 0.71)	<0.001
Model 2	5,578	0.66 (0.55 ~ 0.79)	<0.001
Model 3	5,578	0.66 (0.55 ~ 0.80)	<0.001

Crude model, not adjusted; Model 1, adjusted for age, gender and race; Model 2, Model 1 + marital status, BMI, PIR, education level, smoking status, CVD, PA total time and PA total MET; Model 3, Model 2 + DM and Hypertension; OR, odds ratio.

#### 3.1.3. Associations between NAFLD and serum vitamin C levels (tertiles)

Table 3 shows the association of different concentrations of serum vitamin C with the risk of developing NAFLD. Logistic Regression analysis found that in the four models with different regulatory factors, the risk of NAFLD was lower in the T3 group (≥1.06 mg/dl) than in the T1 group (≤0.69 mg/dl). The T3 risk decreased by 33.0% in crude model without controlled covariates (OR: 0.67, 95% CI: 0.59 ~ 0.77, *p* < 0.001). After adjusting for the covariates, the protective effect of serum vitamin C concentration on NAFLD risk was stable in the T3 group from model 1 to model 3 (Model 1: OR: 0.59, 95% CI: 0.51 ~ 0.67, *p* < 0.001; Model 2: OR: 0.59, 95% CI: 0.48 ~ 0.73, *p* < 0.001; Model 3: OR: 0.59, 95% CI: 0.48 ~ 0.74, *p* < 0.001), but the risk degree of NAFLD decreased slightly compared with model 1.

**Table 3:** Association between serum vitamin C levels and NAFLD (Tertiles), NHANES 2005–2006, 2017–2018.

Vitamin C (mg/dl)	Nonalcoholic fatty liver							
	Crude model		Model1		Model2		Model3	
	OR(95%Cl)	*P*-value	OR(95%Cl)	*P*-value	OR(95%Cl)	*P*-value	OR(95%Cl)	*P*-value
Tertiles
Tertile1	1 (Ref)		1 (Ref)		1 (Ref)		1 (Ref)	
Tertile2	0.8 (0.7 ~ 0.92)	0.001	0.78 (0.68 ~ 0.9)	0.001	0.73 (0.6 ~ 0.89)	0.002	0.72 (0.59 ~ 0.88)	0.002
Tertile3	0.67 (0.59 ~ 0.77)	<0.001	0.59 (0.51 ~ 0.67)	<0.001	0.59 (0.48 ~ 0.73)	<0.001	0.59 (0.48 ~ 0.74)	<0.001
*P* for trend	0.82 (0.77 ~ 0.88)	<0.001	0.77 (0.71 ~ 0.82)	<0.001	0.77 (0.69 ~ 0.85)	<0.001	0.77 (0.69 ~ 0.86)	<0.001

Crude model, not adjusted; Model 1, adjusted for age, gender and race; Model 2, Model 1+marital status, BMI, PIR, education level, smoking status, CVD, PA total time and PA total MET; Model 3, Model 2+DM and Hypertension; OR, odds ratio; CI, confidence interval.

#### 3.1.4. Risk association of serum vitamin C and NAFLD in women and men

Table 4 illustrates the correlation between serum vitamin C levels and the risk of NAFLD in women. In crude model, serum vitamin C has a certain protective effect against NAFLD (OR: 0.60, 95% CI: 0.51 ~ 0.71, *p* < 0.001). When adjusting for covariates such as age and race, the effect increased slightly (OR: 0.54, 95% CI: 0.46 ~ 0.64, *p* < 0.001). However, with more covariates of control, the effect gradually decreased. Of these, serum vitamin C and the risk of NAFLD showed the least effect in model 3 (OR: 0.63, 95% CI: 0.49 ~ 0.80, *p* < 0.001).

**Table 4:** Association of serum vitamin C levels and NAFLD in women.

Models	*n*	Nonalcoholic fatty liver	
		OR (95%CI)	*P*-value
Crude model	2,819	0.60 (0.51 ~ 0.71)	<0.001
Model 1	2,819	0.54 (0.46 ~ 0.64)	<0.001
Model 2	2,819	0.61 (0.48 ~ 0.79)	<0.001
Model 3	2,819	0.63 (0.49 ~ 0.80)	<0.001

Crude model, not adjusted; Model 1, adjusted for age, gender and race; Model 2, Model 1+marital status, BMI, PIR, education level, smoking status, CVD, PA total time and PA total MET; Model 3, Model 2+DM and Hypertension; OR, odds ratio; CI, confidence interval.

Table 5 shows the relationship between serum vitamin C levels and NAFLD in men. In crude model, serum vitamin C showed the weakest protection against the risk of developing NAFLD (OR: 0.78, 95% CI: 0.65 ~ 0.93, *p* = 0,006). However, when adjusting for covariates, the level of protection has slightly increased (Model 1: OR: 0.75, 95% CI: 0.62 ~ 0.90, *p* = 0.002; Model 2: OR: 0.73, 95% CI: 0.56 ~ 0.97, *p* = 0.03; Model 3: OR: 0.73, 95% CI: 0.55 ~ 0.97, *p* = 0.029).

**Table 5:** Association of serum vitamin C levels and NAFLD in men.

Models	*n*	Nonalcoholic fatty liver	
		OR (95%Cl)	*P*-value
Crude model	2,759	0.78 (0.65 ~ 0.93)	0.006
Model 1	2,759	0.75 (0.62 ~ 0.9)	0.002
Model 2	2,759	0.73 (0.56 ~ 0.97)	0.03
Model 3	2,759	0.73 (0.55 ~ 0.97)	0.029

Crude model, not adjusted; Model 1, adjusted for age, gender and race; Model 2, Model 1+marital status, BMI, PIR, education level, smoking status, CVD, PA total time and PA total MET; Model 3, Model 2+DM and Hypertension; OR, odds ratio; CI, confidence interval.

### 3.2. Mendelian randomization study

Under the selection criteria for SNPs, only 8 SNPs remained for analyzing circulating vitamin C on NAFLD from Austee et al. and from FinnGen, respectively. The related excluding SNPs are described in the genetic instrument selection part and Supplementary Table S2. Our findings were unlikely to be affected by weak instruments, according to the F-statistics of the selected SNPs, which ranged from 34 to 420. The detail demonstrations above are presented in Supplementary Table S2.

**Figure 2:**
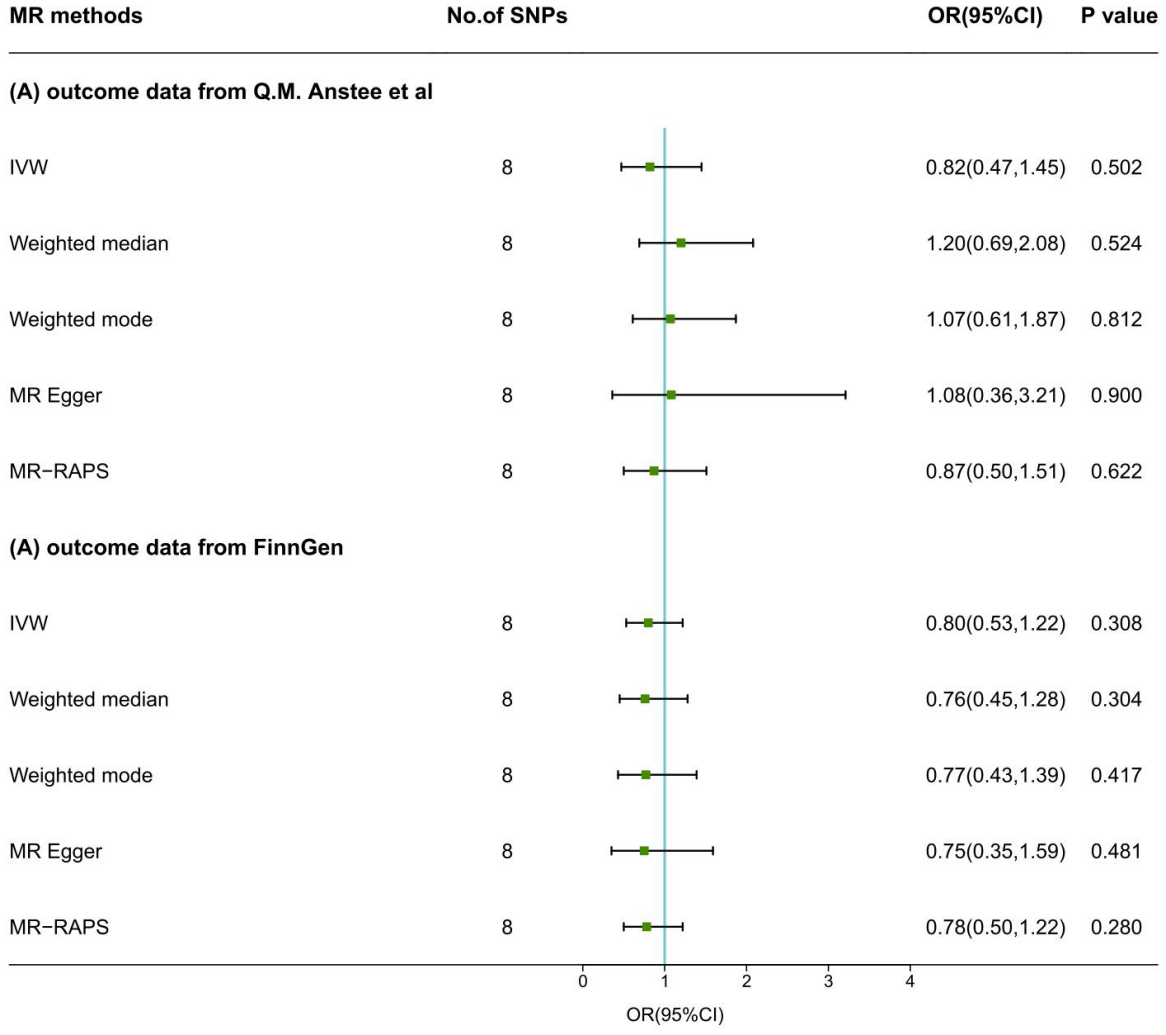
MR results of serum vitamin C levels on NAFLD. SNPs, single-nucleotide polymorphisms; MR, Mendelian randomization; IVW, inverse variance weighted; MR-RAPS, MR-Robust Adjusted Profile Score; OR, odds ratio; CI, confidence interval.

In the IVW model, no causal association was suggested between a one SD change in the log-transformed levels of plasma vitamin C and the risk of NAFLD both in the primary analysis (OR: 0.82, 95% CI: 0.47 ~ 1.45, *p* = 0.502) and the secondary analysis (OR: 0.80, 95% CI: 0.53 ~ 1.22, *p* = 0.308). Results with similar direction and magnitude from two sources were observed in the other four methods (Figure 2; Supplementary Table S3). The leave-one-out analyzes of these results are presented in Supplementary Figure S1, which identified no SNPs strongly influencing the pooled estimates. The scatterplots are presented in Supplementary Figure S2. The forest plots are presented in Supplementary Figure S3, indicating the MR effect size of vitamin C on NAFLD. In the pleiotropic analysis, a slight sign of heterogeneity (Q = 12.42, *p* = 0.088; I^2^ = 39.1%) and no horizontal pleiotropy (Egger intercept = −0.022, *p* = 0.592) was detected in the primary analysis (Table 6). No heterogeneity (Q = 7.34, *p* = 0.394; I^2^ = 0%) and horizontal pleiotropy (Egger intercept = 0.006, *p* = 0.830) was found in the secondary analysis (Table 6).

**Table 6:** Sensitivity analysis between vitamin C and NAFLD.

Outcome	Exposure	Cochran *Q* test			Rucker’s *Q*’ test		Q-Q’		MR-Egger		MR-PRESSO
		*Q* value	*P*	*I* ^2^	*Q* value	*P*	*D*-value	*P*	Intercept	*P*	Global test *P*
NAFLD from Austee et al.	Vitamin C	12.42	0.088	39.10%	11.79	0.067	0.63	0.427	−0.022	0.592	0.129
NAFLD from FinnGen	Vitamin C	7.34	0.394	0%	7.28	0.296	0.06	0.805	0.006	0.830	0.472

## 4. Discussion

In this study, we combined a cross-sectional investigation using the NHANES 2005–2006 and 2017–2018, and a two-sample MR analysis with the summary GWAS data to investigate the relationship between serum vitamin C levels and NAFLD risks. Our observational research results indicated that serum vitamin C concentration were inversely related with the risk of NAFLD especially among females, but MR suggested that this association was not causal.

Observational studies focusing on the protective effect of increased serum vitamin C levels or vitamin C intake on NAFLD were numerous (11, 12, 30–35). However, compared with dietary recall questionnaires, serum vitamin C concentrations can measure physical vitamin C levels more exactly. Published studies have showed an inverse correlation of serum vitamin C concentrations with NAFLD risk (12, 31, 35), which was consistent with our results. It is worthy noted that our findings proved that the serum vitamin C was protective against NAFLD in both women and men, which was conflicting in previous studies reporting nonsignificant results among men (12, 35). The more significant protection effects among females may possibly be a result of the lower levels of mitochondrial oxidative stress and the higher levels of antioxidant enzymes (36).

Clinical trials were relatively deficient focusing on the protective effect of increased serum vitamin C levels or vitamin C intake on NAFLD. A 24-month randomized controlled trial (RCT) by Nobili et al. found that diet intervention and added physical activity were related with a critical improvement in abnormal liver histology and laboratory examination in pediatric NAFLD rather than the pharmacological treatment including alpha-tocopherol plus ascorbic acid (37). Similarly, another 12-month RCT indicated that dietary control and physical exercise can be responsible for a marked improvement of liver work and glucose metabolism in NAFLD children exceeding any antioxidant therapy (38). Nevertheless, a recent RCT suggested that a total of 12 weeks of vitamin C supplementation (particularly 1,000 mg/day) ameliorated liver function and glucose metabolism in NAFLD patients (39). All in all, whether traditional observational research with notable biases from unmeasured or uncontrolled confounders or the three mentioned small-scale RCTs with various study design and measurements can affect the accuracy of results. MR analysis can estimate the lifetime impact of serum vitamin C levels and not the impact at a specific time or a period, with GWAS datasets of large-scale sample. Therefore, it is genetically vital to study the association between serum vitamin C concentration and the risk of NAFLD.

Both the primary and secondary MR analyzes indicated that genetically predisposition to circulating vitamin C was not casually related with NAFLD, whereas our observational study also supported the inverse association between them. The specific relationship of vitamin C on NAFLD is currently unknown, but several potential biological mechanisms exist. On the one hand, as a commonly used antioxidant, vitamin C could reduce the generation of mitochondrial ROS, increase the levels of antioxidant enzymes, and improve the electron transport chain activity found in the liver (40). On the other hand, a study reported that long-term vitamin C deficiency can decelerate the development of NAFLD by inhibiting *de novo* lipogenesis (41). Such results illustrated the double impacts of vitamin C on NAFLD where its anti-oxidative impact may work in restraining NAFLD progression while scarce vitamin C can suppress *de novo* lipogenesis. Consequently, a null association between circulating vitamin C concentrations and the risk of NAFLD in MR studies might result from a counterbalance of anti-oxidative effect and lipid accumulation. The results were also consistent in our sensitivity analyzes, showing the validity of genetic instruments and the stable estimations.

The current study has several strengths. The primary advantage of this study is a large cross-sectional NHANES-based study combined with a two-sample MR analysis. Cross-sectional studies can explore the relationship between serum vitamin C concentration and NAFLD risk from the epidemiological level. MR study addresses the inherent impacts of residual confounding, reverse causality, and measurement errors in conventional epidemiological investigations. Besides, two separate GWAS datasets of NAFLD generated similar results, supporting the robustness of our MR analyzes. There are also some limitations that should be noted. First, because the diagnosis and treatment regimen of NAFLD are not clear, this study only determined hepatic steatosis and fibrosis by CAP in VCTE, and then defined NAFLD. Second, with sex-stratified difference observed in our cross-sectional study, we cannot further explore the sex-specified association in the MR study for the lack of available GWAS data. More studies are needed to investigate the sex-specific associations and confirm our findings between genetically predisposition to serum vitamin C levels and NAFLD risk. Third, this MR study is limited by the comparatively insufficient cases of NAFLD in both two sources. Thus, we cannot exclude weak correlation of genetically determined serum vitamin C levels with NAFLD. Finally, our cross-sectional and genetic data did not come from the same samples, because we employed people of European ancestry for the MR research and members of a multi-ethnic U.S. population for the cross-sectional investigation. To rule out potential confounding variables for population heterogeneity, future work is needed on the same ethnicity.

## 5. Conclusion

In summary, our study did not support a causality between genetically determined serum vitamin C and the risk of NAFLD, even though the cross-sectional study indicated a significant association between them. The observational study results could be biased due to uncontrolled confounders. Therefore, based on the current study, no strong evidence proved that higher serum vitamin C may be help in the prevention or treatment of NAFLD. Further studies with larger cases are required to confirm our conclusions.

## Data availability statement

The original contributions presented in the study are included in the article/Supplementary files, further inquiries can be directed to the corresponding authors.

## Author contributions

J-LG and HW conceptualized the study, analyzed and interpreted the data, and revised and edited the manuscript. J-LG, HW, J-JiY, J-JuY, XT, and W-QH drafted the manuscript. J-LG, HW, R-YX, and G-MH provided advice for the project. W-QH and G-MH provided administrative support. All authors read and approved the final manuscript.

## Conflict of interest

The authors declare that the research was conducted in the absence of any commercial or financial relationships that could be construed as a potential conflict of interest.

## Publisher’s note

All claims expressed in this article are solely those of the authors and do not necessarily represent those of their affiliated organizations, or those of the publisher, the editors and the reviewers. Any product that may be evaluated in this article, or claim that may be made by its manufacturer, is not guaranteed or endorsed by the publisher.

## Footnotes

^1^ http://www.phenoscanner.medschl.cam.ac.uk/
