# Supplementary material for: Serum vitamin C levels and risk of non-alcoholic fatty liver disease: results from a cross-sectional study and Mendelian randomization analysis
Source: Front Nutr. 2023 May 12;10:1162031. doi: 10.3389/fnut.2023.1162031 (PMC10213341; doi:10.3389/fnut.2023.1162031)

**Supplementary text**

Covariates

To build up the adjustment model, the following covariates would be added based on previous studies of NAFLD. Categorical variables included race (Mexican American, non-Hispanic black, non-Hispanic white, other Hispanic, other race–including multiracial), marital status (married, unmarried), body mass index (BMI) (25, 25–29.9, ≥30 kg/m^2^), education level (less than high school, high school graduation, college or above), smoking status (never = smoked <100 cigarettes in life, former = smoked <100 cigarettes in life and smoke not at all now, now = smoked moth than 100 cigarettes in life, and smoke some days or every day), cardiovascular diseases (CVD) (“Yes” or “No”), diabetes mellitus (DM) (“Yes” or “No”) and hypertension (“Yes” or “No”). And there are five continuous variables: age, poverty income ratio (PIR), physical activity (PA) total time, PA total metabolic equivalent (MET) and the serum concentration of vitamin C. CVD is defined as any diagnosis of congestive heart failure, coronary heart disease, angina, heart attack, or stroke. When residents asked, “Has a doctor or other health professional ever told you that you have congestive heart failure/coronary heart disease/angina/heart attack/stroke?” and those who answered "yes" to either question were included in the general cardiovascular disease group of our study. The sample will be considered hypertensive if a physician diagnoses it as hypertensive and prescribes hypertension medication or measures a blood pressure of 140/90 mmHg, ambulatory blood pressure monitoring: mean blood pressure ≥ 130/80 mmHg within 24 h, daytime ≥ 135/85 mmHg, at night ≥ 130/80 mmHg. The following criteria were used to determine whether a participant was DM: (1) had been diagnosed with all types of diabetes; (2) fasting blood glucose >7.0 mmol/L; (3) random blood glucose or 2-h The oral glucose tolerance test (OGTT) blood glucose >11.1 mmol/L; (4) had used diabetes medications or insulin.

**Figure S1**. Leave one out analyses of serum vitamin C on NAFLD (a: NAFLD-Anstee et al GWAS; b: NAFLD-FinnGen GWAS)


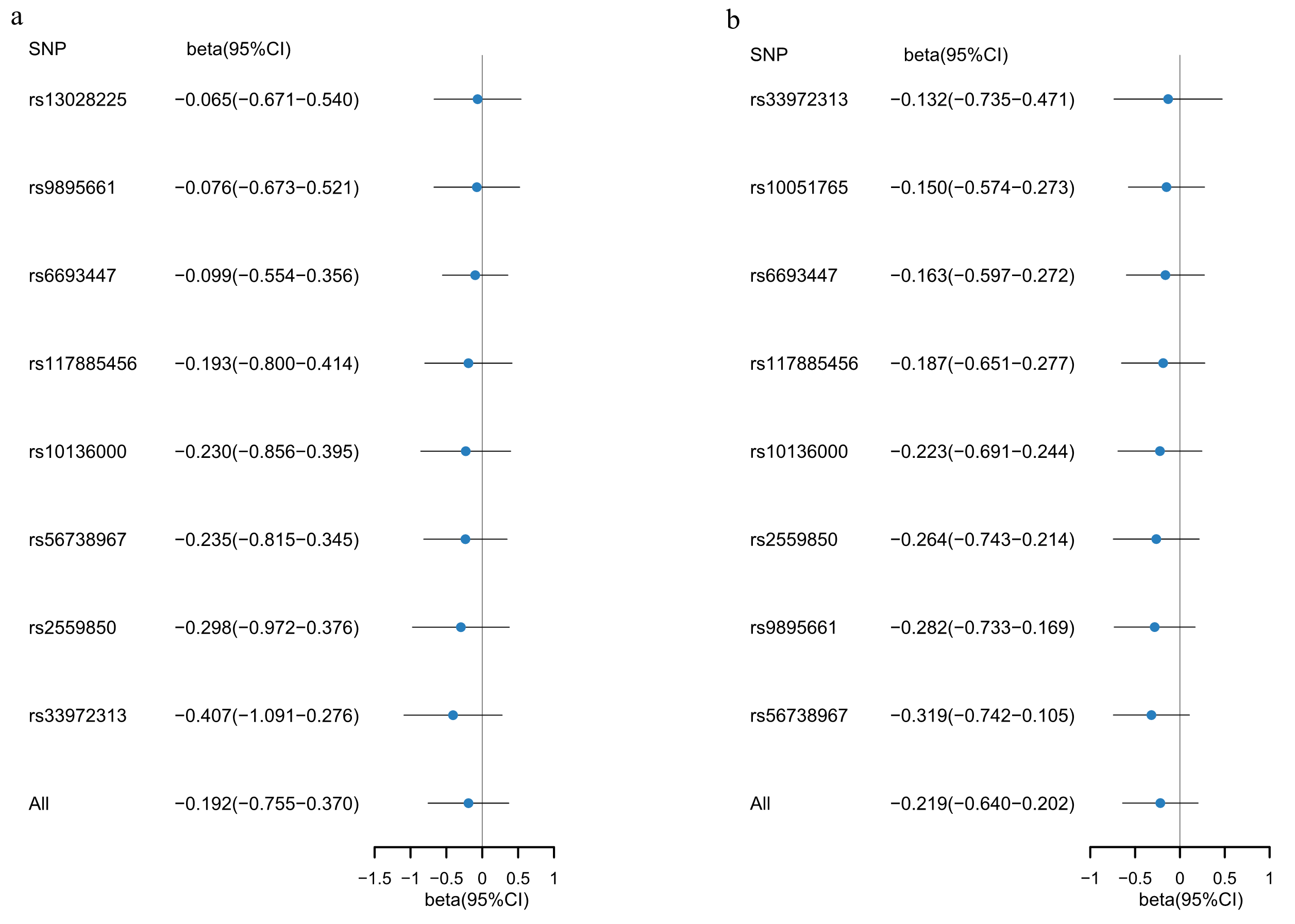


**Figure S2**. Scatterplots of serum vitamin C on NAFLD (a: NAFLD-Anstee et al GWAS; b: NAFLD-FinnGen GWAS)


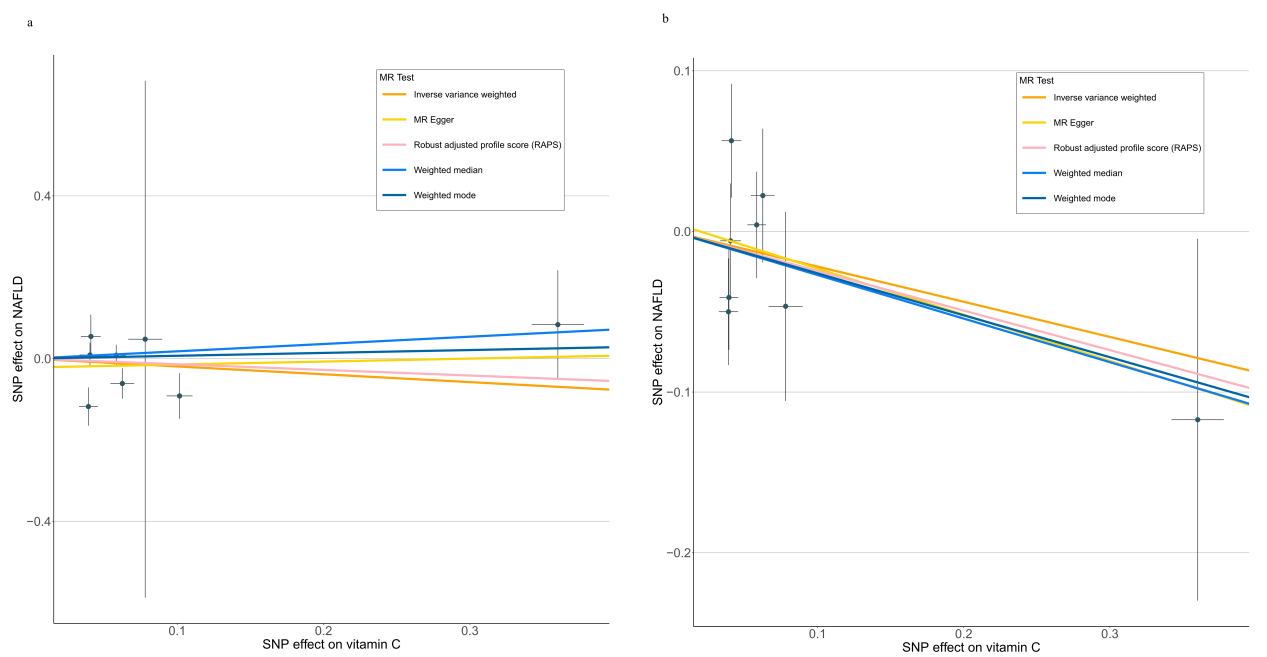


**Figure S3**. Forestplots of serum vitamin C on NAFLD (a: NAFLD-Anstee et al GWAS; b: NAFLD-FinnGen GWAS)


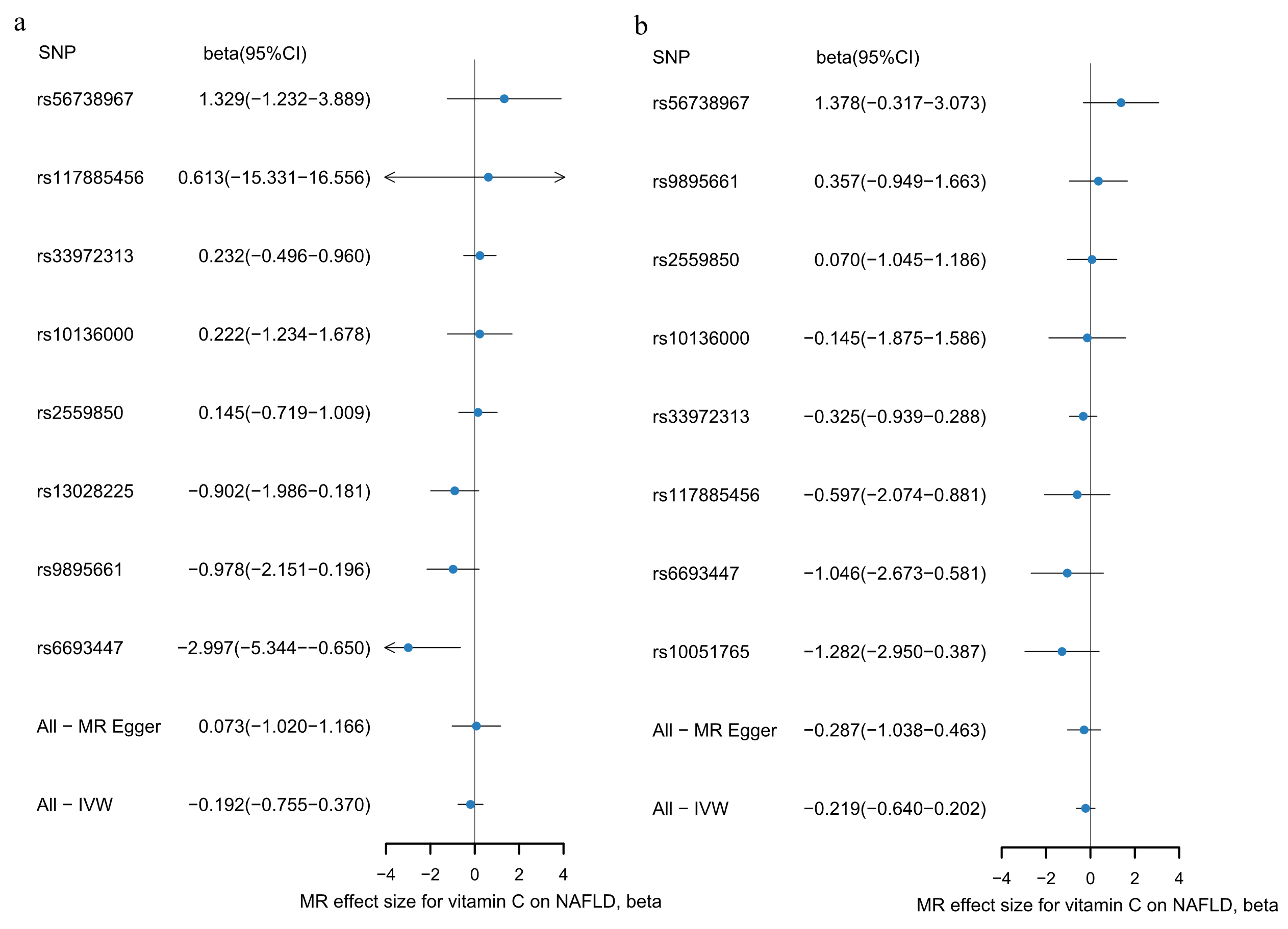

Supplement: Supplementary file 1 [file Data_Sheet_1.docx]
